# Exploring the quality of life of pediatric trainees: Mixed-method explanatory design

**DOI:** 10.12669/pjms.40.11.6853

**Published:** 2024-12

**Authors:** Hina Mumtaz Hashmi, Asma Pethani, Sana Saeed, Ali Faisal Saleem

**Affiliations:** 1 Hina Mumtaz Hashmi, MBBS, FCPS, MRCPCH. Senior Instructor, Department of Pediatrics and Child Health, Aga Khan University Hospital, Stadium Road, Karachi, Pakistan; 2 Asma Pethani, BDS, MSc (HPM) Research Coordinator, Department of Pediatrics and Child Health, Aga Khan University Hospital, Stadium Road, Karachi, Pakistan; 3 Sana Saeed, MBBS, FCPS, MHPE. Assistant Professor, Department of Pediatrics and Child Health, Aga Khan University Hospital, Stadium Road, Karachi, Pakistan; 4 Ali Faisal Saleem, FCPS, MSC (Clin Res), PDIP PID (OXF) Associate Professor, Department of Pediatrics and Child Health, Aga Khan University Hospital, Stadium Road, Karachi, Pakistan

**Keywords:** Burnout, Compassion fatigue, Compassion satisfaction, Pediatric residents, Professional quality of life, Secondary traumatic stress

## Abstract

**Objective:**

Healthcare workers are at a higher risk of burnout. The aim of the study was to explore the professional quality of life (QOL) i.e. {Compassion Satisfaction (CS), Burnout (BO) and Secondary Traumatic Stress (STS)} and association of different trainee factors on QOL amongst Pediatric specialty trainees at a private tertiary care hospital in Karachi, Pakistan.

**Methods:**

By using a mixed methods explanatory design, pediatric trainees experience of the prior year was measured in 2019 and 2020 through a predefined scale at Aga Khan University Hospital, Professional Quality of Life (ProQOL), followed by focused group discussions. Mean sums of CS, BO and STS were the primary measures assessed and the associations of different trainee factors were to explore experiences of Pediatric trainees towards the residency program, themes and subthemes were created after focused group discussions

**Results:**

We surveyed a total of 118 pediatric responses (n=60 and n=58) during two years.Trainees from both years showed similar moderate level for mean sum of scores for all three components; CS, BO and STS. No significant association was observed with gender, place of living or year of residency. Opinions and perceptions of the trainees in qualitative analysis revealed both positive and negative impacts on the quality of life of the trainees.

**Conclusion:**

This study delves into the professional quality of life and offers valuable understanding into the specific events encountered by trainees.

## INTRODUCTION

Healthcare workers are at a higher risk of overall burnout as compared to the general population (40% vs 28%).^1^ Similarly, healthcare workers have a lower satisfaction of work life integration (40% vs 61%) and a higher rate of emotional exhaustion (36% vs 25%) as compared to the general population.^1^ Some specialties demonstrate higher burnout like emergency settings.^2^ Symptomatology associated with burnout includes depression, disconnect, becoming judgmental and disrupted beliefs, whereas risk factors include a first degree relative with depression (57%), living away from family (73%), and history of psychiatric illness (57%).^3^,^4^

Professional quality of life is the quality one feels about their work as a helper. The positive and negative aspects of work influence professional quality of life. However, the exemplary CS and Compassion fatigue (CF) amongst helpers such as teachers, police officers, healthcare professionals etc.; is explained in terms of the good things and bad things associated with helping others who experience suffering.^5^ The specific form of BOthat directly affects those involved in care-giving professions has been termed “compassion fatigue”.^6^

BO relates to fatigue and frustration whereas STS relates to negative feelings from experience of work-related.^5^-^7^ CS explains the ‘resiliency of the human spirit’, being a possible factor balancing the effects and risks of CF.^8^ On seeing recovery or a ‘change for the better’ amongst patients and families, doctors feel a sense of return.^9^ The environment plays a major role in continuity of compassion amongst trainees, either directly or indirectly.^10^ To assess all three parameters, CS, BO and STS, a ProQOL scale has been created in the past and has been pretested and used by multiple authors.^5^ Literature review using the ProQOL scale, revealed low CS (24%) as compared to BO (37%) and STS (72%) among otolaryngology residents.^11^

Similarly, ProQOL amongst aid workers showed that BO was positively related to general distress and STS, and negatively related to CS.^12^ Studies using different tools for measuring CF and CS have been done amongst Pediatric Health care providers, revealing the impact on professional performance of the health care workers with a focus on targeting interventions towards increasing CS.^13^,^14^ It is crucial to gain a better understanding to the extent of which trainees are affected by conditions such as BO and CF as this is critical for cultivating a positive and nurturing practice environment for all trainees.^5^

It is also imperative to understand the support needed, that enables them to be more proactive while working in an already stressful environment. There are very few studies that assessed the BO, CF, and STS from resource limited settings,^12^ and as per our knowledge there has been no study conducted in Pakistan specifically for Pediatric trainees analyzing their ProQOL. In this study, we aimed to;

- Measure the CS, BO and STS amongst pediatric trainees using the ProQOL tool.
- Determine the association of different trainee factors on QOL.
- Explore the experiences of Pediatric Trainees at two different time points about their training program at a University Hospital in Karachi.

## METHODS

A mixed-method explanatory design, comprising of three-phases; (two quantitative for the year 2018 and 2019 conducted in 2019 and 2020 followed by a qualitative phase in 2020) was performed amongst all pediatric trainees at Aga Khan University Hospital. Non-probability consecutive sampling was used to collect data from a total of 118 pediatric trainees (60 from 2018 and 58 in 2019) of which thirty eight residents responded in both years to demonstrate any impact of maturation on scores of quality of life. A self-administered questionnaire filled via google docs was used and convenience sampling was done for qualitative data collection. Aga Khan University hospital (AKUH) is one of the biggest tertiary care hospitals of Karachi, Pakistan, covering a population of ~ 18 million people. The Department of Pediatrics initiated the residency training program in the year 1986, one year after the inauguration of the first postgraduate clinical training program with an origin from four seats to a current extension of 58. This shows the competing interest of our medical graduates for this sub specialty. The trainees here are exposed to a vast variety of rotations.

### Data Collection Tool:

After permission, Professional Quality of Life scale (ProQOL-Version-5) by Stamm was utilized to collect data on CS, BO, and STS.^5^ The scale has been used globally with well-established construct validity. It covers mainly three measures with a total of 30 items and for each sub-scale, total score is reported as low, moderate or high.

### Sampling:

A non-probability convenience sampling was done

### Ethical Approval:

The study was commenced after the approval from Ethical Review Committee (ERC # 662) of AKUH dated January 29, 2019.

The study flow process in Fig-I represents the three phases of the study. The first phase of Quantitative data collection was from February - March 2019 for the work experience of 2018. Results were analyzed according to the three measures, CS, BO, and STS. Analysis was shared with the program but no intervention was taken. The second phase took place from February - March 2020 collecting data of 2019 including new residents as well. Results were analyzed according to the three measures, CS, BO and STS for this year and compared with previous data from phase-1. Based on score, data was divided into low, moderate and high scores.

**Fig.1:**
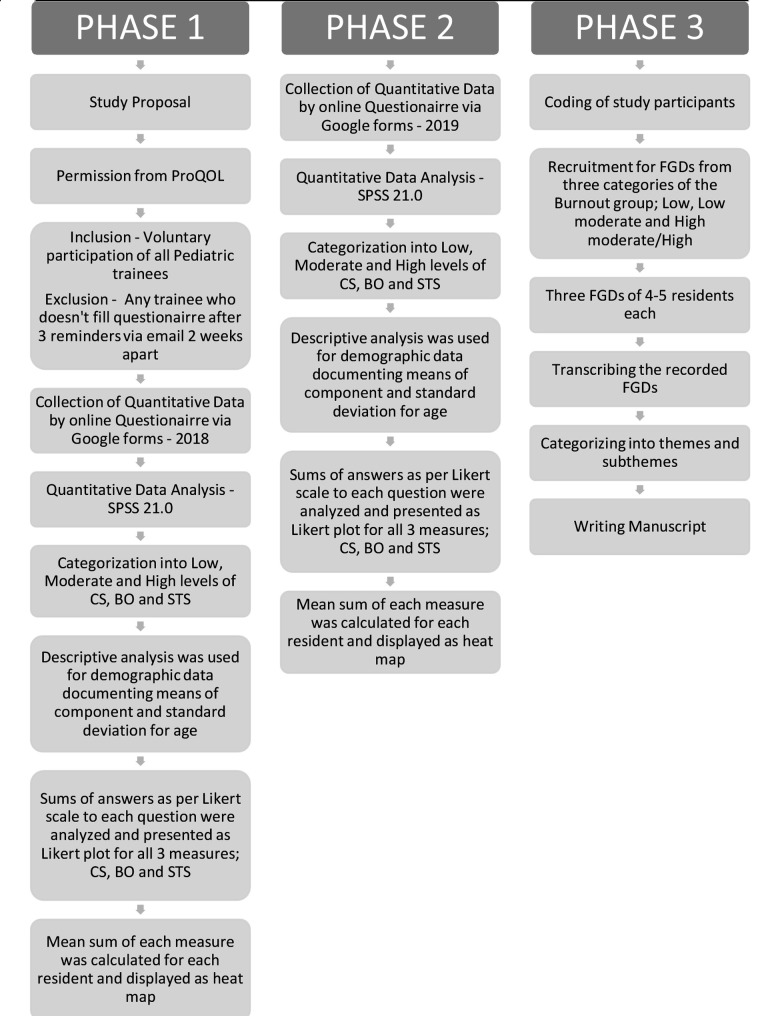
Study Flow Process showing methodology. ProQOL: Professional Quality of Life; CS: Compassion Satisfaction; BO: Burnout; STS: Secondary Traumatic Stress; FGD: Focused Group Discussion.

After quantitative data, all respondents were mailed and invited to participate in the three FGD (Focused group discussions) according to scores of Burnouts; one for low, one for low moderate and the third for high moderate and high combined, but participants were unaware of their score. Three FGDs were conducted with a total of 13 respondents (five respondents in group one, four in groups two and three). Verbal consent was taken prior to the FGDs. Interviews comprised of eight open ended questions focusing on resident training experience, the work environment, their perception on quality of life and the different measures used in the PROQOL scale; Compassion satisfaction, Compassion Fatigue, Burnout and Secondary Traumatic Stress. Also their ideas and suggestions regarding improvement in the training program were noted.

### Ethical considerations:

Participant’s information was presented in a coded manner and no personal identifiers were shared. Data confidentiality and anonymity was maintained in reports or publication of the study.

### Data analysis:

Data analysis was carried out using SPSS (Version 21) for quantitative data. Frequency distributions of all the variables were checked for outliers, missing data, and typing errors. Summary statistics, including the computation of means, standard deviations (SDs), frequency counts, and percentages of all demographic data and ProQOL measures (CS, BO, and STS) was performed (Table-I). Pearson correlation and paired t-tests were done for all three measures for thirty-eight trainees from 2018 who continued to be part of training in 2019.

**Table-I:** Frequency analysis of Demographic Data of Pediatric Trainees and ProQOL Measures; 2018 and 2019.

Total Number of Pediatric Trainees = n		2018 (n=60)	2019 (n=58)
Year of Residency – n (%)	Year 1	10 (17%)	20 (35%)
	Year 2	18 (30%)	10 (17%)
	Year 3	17 (28%)	14 (24%)
	Year 4	15 (25%)	14 (24%)
Age (years)	Median	28	28
	<30 years	49 (82%)	43 (74%)
	>/= 30 years	11 (18%)	15 (26%)
	Standard Deviation	1.689	2.094
Gender – n (%)	Female	44 (73%)	49 (85%)
Marital Status	Married	16 (27%)	15 (26%)
Years in Clinical Practice	Mean	4	4
Working hours/week	Mean	90	90
***Hosteler – n (%)***		15 (25%)	10 (17%)
Compassion Satisfaction	Mean Sum	37	36
	Level	Moderate	Moderate
	Standard Error	0.735	0.836
	Max Sum	49	46
	Min Sum	20	17
Burnout	Mean Sum	29	29
	Level	Moderate	Moderate
	Standard Error	0.638	0.659
	Max Sum	40	40
	Min Sum	18	19
Secondary Traumatic Stress	Mean Sum	30	29
	Level	Moderate	Moderate
	Standard Error	0.713	0.802
	Max Sum	45	42
	Min Sum	13	14

Moderate scores were further subdivided into low and high moderate with a cutoff at score 32. Manual content analysis was performed on qualitative data. COREQ-23 guidelines were used to model and structure the Qualitative component.^15^ Categories were identified using manual coding and major patterns and trends in responses were reported as themes and subthemes (Table-II).

**Table-II:** Themes and sub-themes in Qualitative analysis.

THEMES	SUB-THEMES
Impact of academic structure	1. Academic program in trend
	2. Bedside teaching
	3. Time constraint
	4. Exam Orientation
Influence of training/work environment	1. Rotation Exposure
	2. Future prospects
	3. Learning
	4. Workload
	5. Impact
	6. Appeals
Effect of organizational Structure	1. Protocols
	2. Documentation
	3. Demand
	4. Senior Behavior
	5. Ownership
Quality of life; understanding and perception	1. Understanding
	2. Impact on married trainee’s 3.Effect of environment
	4. Compassion Satisfaction
	5. Compassion Fatigue
	6. Burnout
	7. Secondary Traumatic Stress
	8. Work-life balance
	9. Driving Factor
Strategies to enhance the quality of life	1. Time adjustment
	2. Behavioral modification
	3. Resourcing
	4. Academic Learning
	5. Certainty
	6. Extracurricular activities

## RESULTS

### Phase-I and II: Quantitative-conducted in 2019 and 2020:

A 100% response was observed to the questionnaire in both phases. In both years, a similar trainee median age was noted at 28 years (Range 25-36 years), with a female predominance (n=44, 73% and n=49, 85%) and a similar proportion of marital status, 73% vs 74% being single respectively. The percentage of hostellers were 25% and 17%, respectively in 2018 and 2019 (Table-I).

Mean sum for all three scales were similar in both years with the mean level being at moderate (Table-I). Descriptive analysis for each level of the scale was done for all three measures. CS was noted to be mostly in high moderate scores whereas both BO and STS levels were calculated to be at low moderate levels for trainees in both years.

The Likert chart showed “Often” as the major response for questions in the CS measure for both years (2019, mean 39% and 2020, mean 44%). As for the questions corresponding to BO and STS, most marked options varied from rarely - Often depending on individual questions.

Heat map demonstrated CS for both years yielding scores towards higher levels whereas BO and STS were more towards lower levels. Cross-tabulation of PROQOL measures showed that in the 2019 survey, 52% of total trainees had both low moderate BO and STS as compared to 33% trainees in 2020. Whereas for CS to BO, it was noted that trainees with Low Moderate levels of burnout had High Moderate levels of compassion satisfaction; 48% vs 34% in both years. For CS and STS; trainees with high moderate levels of CS showed low moderate levels of STS; 45% trainees in 2019 which decreased to 26% in 2020.

No statistically significant correlation was observed between gender and place of living with the ProQOL measures. Correlation between measures of trainees from 2019 who were reassessed in 2020, found to be statistically significant with a p-value of < 0.05.

### Phase III: Qualitative:

**Fig.2:**
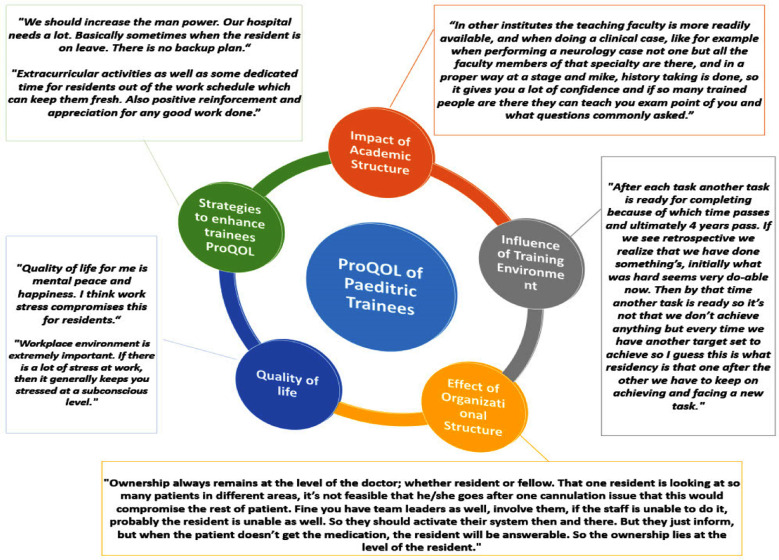
Themes identified for Professional Quality of life of Pediatric trainees.

Five core themes headings were generated from the data analysis. **(1)** Academic structure, **(2)** Training/work structure, **(3)** Organizational structure, **(4)** Quality of life, and **(5)** Strategies. Within each core theme were multiple subthemes further describing the categories (Table-II, Fig.2) Workplace interventions include education about burnout, workload modifications, increasing the diversity of work duties, stress management training, mentoring, promoting interpersonal professional relations, meditation, counselling, exercise, emotional intelligence training, and wellness workshops for the trainees. Occupational interventions in work settings can also help improve emotional and work-induced exhaustion. 5

## DISCUSSION

The study identified a cumulative low moderate score for burnout and secondary traumatic stress amongst pediatric trainees. We find a few pediatric trainees in high moderate burnout (22% and 24%) as compared to 59% of pediatric residents at a tertiary care hospital in Lahore indicating a possible difference in the overall quality of life.^16^ Despite burnout many trainees were very compassionate with high moderate to high scores (78% and 67%) and willing to deliver and be trained despite being in stressful situations like the influence of the training environment including mostly documentation, workload and demand.

In a similar study addressing work life balance amongst residents of different specialties, 60% residents felt their jobs had negatively affected their private lives.^17^ The “perfect” work environment in terms of managing stress and trauma is one that combines a high CS with a low BO and STS.^5^ Some In studies a higher score of BO and STS was possibly sue to emotional exhaustion of the healthcare workers, poor working condition and working in government organizations.^18^ Theoretically, a high burnout amongst health care workers leads to higher chances of medical errors.^5^

In this study, many concerns were identified in the qualitative analysis of the trainee perspective on the quality of life and impacts from a work-place. A poor work environment contributes to CF whereas at the same time a person could feel CS that they could help others despite that poor work environment as was seen in our study.^5^ On the contrary, different structural aspects identified via the qualitative analysis, the organizational structure was mostly highlighted. Trainees perceived that most of their time is spent providing services to patients rather than case learning.^19^

Literature shows that faculty members and trainee perceptions are often not aligned.^20^ In contrast to our study, the trainee and faculty relation is person dependent with some being very positive and others creating an environment of negativity. To help improve the work life balance, 61.5% of trainees suggested a need for a friendlier faculty.^17^ Also, the trainee workload seems to have a major impact on the trainee’s quality of life. A hypothesis states that more time for reflection, fewer breaks, and greater interaction with faculty may explain the observed differences in resident performance because of the reduction in workload.^21^

Patient census is commonly used as a rough approximation to determine trainee workload as it does not account for patient complexity. A study in a similar setting correlates with this, with a 55% of high degree burnout due to patient census.^16^. Furthermore, work-related stress must be considered which includes interruptions like pages, communication between team members, protocols and documentation.^16^,^22^ A time-motion study, showed that one-third of residents’ time was spent in activities of marginal educational value, such as test ordering, tracking down results, and ancillary tasks (phlebotomy or transportation) similarly expressed by our residents.^23^

Trainees in this study felt that maximum time was spent in arranging “things” for patients and organizational work like documentation. Documentation necessities also add to the burden on trainees and other documentation activities might not augment learning as per resident opinion. Documentation was also positively highlighted as a part of the learning process in a trainee’s work experience.^21^ Different studies have focused on developing physician work hour regulations through the process of a coordinated agenda addressing resident concerns specifically duty hours to facilitate the spread of best clinical practice and optimizing resident education with an ultimate focus on patient care and its positive outcomes.^24^

### Limitation:

Targeting only one residency specialty. Results are not generalizable for the rest of the country. Small group of respondents in qualitative analysis.

## CONCLUSION

The study identified a cumulative low moderate score for burnout and secondary traumatic stress amongst pediatric trainees. The apparent stresses are not unique to Pediatric Trainees and represent an indicator for individual growth. Residency programs can be more helpful to trainee residents by giving more support. A combination of both individual and workplace interventions including stress management programs will ultimately have a good impact in reducing the burnout scores and teach trainees how to cope better with stressful events. These efforts may lead to develop more effective physicians with competent human care towards their patients.

### Future implications:

Nap hours or sleep pods can be introduced to help tired health care workers take a break as being trialed in NHS hospitals.^25^ Clinical departments should shift from quality assurance towards a continuous improvement of state of the mind for a more positive learning environment.^26^

### Disclosure:

The authors would like to acknowledge the ProQOL team for permitting to use this scale and the respondents that freed up their time to participate in this study.

### Authors’ contribution: HMH:

Contributed to the study design, questionnaire design, acquisition of data, data interpretation, drafting the article, literature search and provided feedback through critical manuscript review. **AP:** Contributed to the literature search, data collection, analysis, and interpretation. **SS:** Contributed to the study concept, questionnaire design, data interpretation, and provided feedback through critical manuscript review. **AFS:** Contributed to the study concept, data interpretation and analysis, and provided feedback through critical manuscript review. All authors have read the final version and are responsible and accountable for the accuracy and integrity of the work.
